# Quasi-3D Hyperbolic Shear Deformation Theory for the Free Vibration Study of Honeycomb Microplates with Graphene Nanoplatelets-Reinforced Epoxy Skins

**DOI:** 10.3390/molecules25215085

**Published:** 2020-11-02

**Authors:** Hossein Arshid, Mohammad Khorasani, Zeinab Soleimani-Javid, Rossana Dimitri, Francesco Tornabene

**Affiliations:** 1 Department of Mechanical Engineering, Qom Branch, Islamic Azad University, Qom 3749113191, Iran; Arshid@ut.ac.ir; 2 Department of Basic and Applied Sciences for Engineering, Faculty of Civil and Industrial Engineering, Sapienza University, 00161 Rome, Italy; Mohammad.Khorasani94@hotmail.com; 3 Department of Solid Mechanics, Faculty of Mechanical Engineering, University of Kashan, Kashan 8731753153, Iran; As.Javid71@gmail.com; 4 Department of Innovation Engineering, Università del Salento, 73100 Lecce, Italy; rossana.dimitri@unisalento.it

**Keywords:** graphene nanoplatelets, honeycomb structures, modified couple stress theory, quasi-3d hyperbolic shear deformation theory, sandwich structures, vibration analysis

## Abstract

A novel quasi-3D hyperbolic shear deformation theory (QHSDT) with five unknowns is here employed, together with the Hamilton’s principle and the modified couple stress theory (MCST) to analyze the vibrational behavior of rectangular micro-scale sandwich plates resting on a visco-Pasternak foundation. The sandwich structure features a Nomex or Glass phenolic honeycomb core, and two composite face sheets reinforced with graphene nanoplatelets (GPLs). The effective properties of both face sheets are evaluated by means of the Halpin-Tsai and extended rule of mixture (ERM) micromechanical schemes. The governing equations of the problem are derived by applying the Hamilton’s principle, whose solutions are determined theoretically according to a classical Navier-type procedure. A parametric study checks for the effect of different material properties, length-scale parameters, foundation parameters and geometrical properties of the honeycomb cells, and the reinforcing GPLs, on the vibration response of the layered structure, which can be of great interest for many modern engineering applications and their optimization design.

## 1. Introduction

In the last decades, lightweight mechanical components and layered structures have increased the attention of many researchers and scientists, due to the increased demand in modern engineering, together with a possible reduction in their production cost. Among them, sandwich structures can be regarded as subset of multilayered composite structures consisting of outer facings and a soft core in-between, including foam, honeycomb, corrugated core, various bio-inspired cores, etc. The choice of sandwich materials depends on the structural functionality as well as on the lifetime loading, availability and cost. For example, Graphite-epoxy and carbon-epoxy multilayered facings are typically used in aerospace applications, whereas glass-epoxy or glass-vinyl ester are adopted in civil and marine layered structures. At the same time, the core of sandwich aerospace structures is often made of aluminum or Nomex honeycomb, whereas, a closed-cell or open-cell foam represents the typical core choice in civil engineering, instead of a balsa core, usually applied in ship sandwich structures. As far as the honeycomb sandwich-type plate is concerned, the adhesive bonding between the honeycomb core and face sheets is the responsible for the load transferring among the sandwich constitutive parts. In such a context, one of the pioneering works on the topic is represented by Ref. [1], where the authors studied the vibrational behavior of sandwich beams with a honeycomb core [1]. In 2004, different vibration tests were performed experimentally by Yanfeng and Jinghui [2] to study the vibration transmissibility and shock-absorbing properties of the honeycomb thin plates, while computing their damping ratios and highest frequencies of vibration. From a theoretical and numerical perspective, a comprehensive review of studies on sandwich structures is mentioned in the following, covering the more recent developments on the topic. Li and Jin [3] applied a third-order shear deformation plate theory (TSDT) and classical plate theory (CPT) to examine the free vibration of rectangular plates with a honeycomb structure, whereas a semi-analytical approach was suggested in [4] for the bending, buckling and free vibration analysis of sandwich panels with square-honeycomb cores. At the same time, the influence of the skin/core debonding phenomena on the overall vibrational behavior of sandwich plates was analyzed by Burlayenko and Sadowski [5], whose results helped to address sandwich plates non-destructive damages. In line with this work, a wavelet analysis has been recently applied by Katunin [6], to detect and identify possible damages in sandwich structures and their effect on the global vibrational structural response. The sensitivity of the vibration response of a honeycomb core structure to random geometrical or mechanical irregularities was also outlined theoretically by Mukhopadhyay and Adhikari [7]. A novel method was proposed by Duc et al. [8] to study the vibrational response of sandwich cylindrical panels with a honeycomb core, based on the first-order shear deformation theory (FSDT), fourth-order Runge-Kutta method and Galerkin method. Among the most recent solutions of increasing the intrinsic damping properties of sandwich structures, Piollet et al. [9] proposed the use of entangled cross-linked fibers as core materials within sandwich beams and performed different steady-state tests for different excitation levels to study their high-damping and nonlinear vibration response. Moreover, Kumar and Renji [10] studied the acceleration response and natural modes of sandwich panels with a honeycomb core subjected a diffused acoustic field, developing a methodology to estimate their strain field in low frequency modes, based on the acceleration response. A novel model based on the differential quadrature method (DQM) was successfully proposed by Sobhy [11] to study the coupled hygrothermal bending response of functionally graded (FG) graphene platelets/aluminum sandwich-curved beams equipped by a honeycomb core. A numerical and experimental investigation based on a classical finite element approach and imaging correlation method was also performed by Li et al. [12] for the study of the dynamic response of shallow sandwich arches with aluminum face sheets and auxetic reentrant hexagonal metallic honeycomb core under a localized impulsive loading, providing useful data and results for the honeycomb cells deformation [12]. In 2017, Chen et al. [13] examined the nonlinear mechanical behavior of a sandwich structure. Their model was made of FG porous layer reinforced by graphene nanoplatelets (GPLs). Moreover, Karimiasl et al. [14] studied the nonlinear vibration behavior of multiscale nanocomposites nanoshells, resting on an elastic foundation, and subjecting to a hygrothermal environment. Furthermore, in 2019, the instability characteristics of a magnetorheological (MR) fluid core patched to two piezoelectric FG-GPLRC face sheets were investigated by Eyvazian et al. [15], while proving the positive effect of magnetic field on the system’s mechanical behavior. More recently in 2020, Torabi and Ansari [16] hired the Mindlin’s plate model and the phase-field approach to have a throughout comprehension of the vibration response for cracked FG GPL-RC plates with stationary cracks. 

The large benefits of sandwich structures and their mechanical performances, have increased the interest of the scientific community to develop even more accurate theories for their study. For example, an improper definition of a mechanical parameter, even at small scales, can cause a meaningful variation in the acquisition of results, with deleterious effects on the overall performance of sensitive systems, as aircraft and space vehicles. This makes extremely important the use of accurate theories, where the proper definition of the mechanical parameters is mandatory to obtain reliable results. In such a context, many works from the literature have applied CPTs, FSDTs, or higher-order-theories (HSDTs) for the study of plate and shell structures even with complicated materials and geometries. For example, Khoa et al. [17] applied a HSDT to examine the vibration response of FG carbon nanotubes (CNTs) reinforced composites cylindrical shells in thermal environment. The same problem was also studied by Ibrahim et al. [18], according to FSDT, and coupled with thermal conditions. Li et al. [19] used CPT to model clamped honeycomb sandwich panels to study the nonlinear forced vibrational response. Many further applications of the HSDT to coupled problems of sandwich panels and shell structures can be found in [20,21,22,23,24,25,26,27,28]. A valid theoretical alternative to handle the plate structures is represented by the quasi-3D hyperbolic shear deformation theory (QHSDT) which accounts for both transverse shear and normal deformations and satisfies the zero traction boundary conditions on the plate surfaces without using any shear correction factor. In QHSDT the number of unknown functions involved in displacement field is only equal to five, instead of six or more unknowns required by the other shear and normal deformation theories. The computational efficiency of this method was recently verified in Refs. [29,30,31]. Inspired by these few pioneering works from the literature, in the present paper we propose a QHSDT to study the free vibration response of sandwich structures with a honeycomb core resting on a visco-Pasternak foundation. The governing equations of the problem are derived from the Hamilton’s principle and solved in closed form via the Navier’s method. The analytical solutions from our formulation are verified with those reported in literature, where a parametric investigations aims at determining the effect of the material variation, GPLs gradient index and dispersion patterns, geometry, internal cells angle, or thickness of layers on the natural frequencies for the selected sandwich structure. 

## 2. Theoretical Formulation

**Figure 1:**
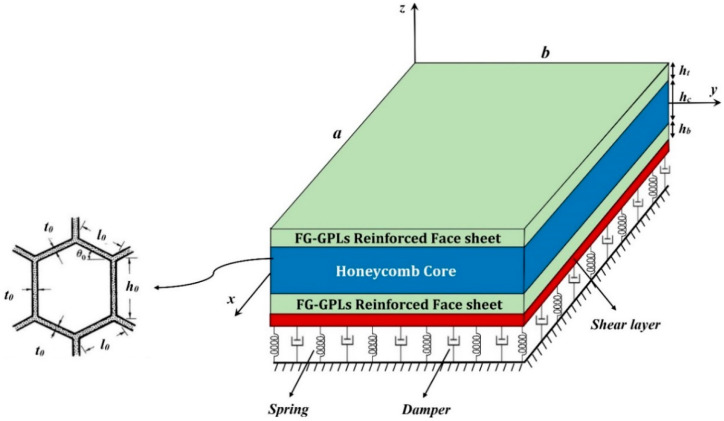
Geometrical model of sandwich structure.

Consider a rectangular sandwich plate with thickness *h*, length *a*, width *b*, as illustrated in Figure 1, together with the reference coordinate system (*x*, *y*, *z*). The sandwich structure is immersed within a visco-Pasternak elastic foundation, and it is made of a honeycomb core with thickness *h_c_* and two composite face sheets with thickness *h_t_* and *h_b_* at the top and bottom side, respectively. This means that the total thickness of the structure is *h* = *h_c_* + *h_t_* + *h_b_*.

In the current study, a QHSDT is adopted to define the position of an arbitrary point in the micro-model. The major advantage of using such a displacement field is that the problem is not limited to plane-strain conditions (i.e., *ε_zz_* ≠ 0), as typically occurs in the other 2-D theories such as FSDT, that could cause possible discrepancies between the theoretical and experimental results. Based on a QHSDT, the displacement field is defined as [32]

(1) $$U(x,y,z,t)=u(x,y,t)-z\frac{\partial }{\partial x}w_{b}(x,y,t)-f(z)\frac{\partial }{\partial x}w_{s}(x,y,t),\;V(x,y,z,t)=v(x,y,t)-z\frac{\partial }{\partial y}w_{b}(x,y,t)-f(z)\frac{\partial }{\partial y}w_{s}(x,y,t),\;W(x,y,z,t)=w_{b}(x,y,t)-w_{s}(x,y,t)-w_{st}(x,y,z,t)$$

where *u* and *v* stand for the displacement components along the *x* and *y* directions, respectively; *w_s_*, *w_b_* and *w_st_* are the transverse displacement components due to bending, shear and stretching effects, respectively, with

(2) $$w_{st}\left(x,y,z,t\right)=g(z)\varphi \left(x,y,t\right)$$

In the last relation, *φ* is an additional displacement variable that accounts for the effect of normal stress; *g*(*z*) and *f*(*z*) are expressed by the following functions [29]

(3) f(z)=((h/π)sinh(πz/h)−z)/(cosh(π/2)−1),

(4) $$g(z)=1-f^{\prime }\left(z\right)$$

where *f*’(*z*) denotes the first derivative of function *f* with respect to *z*. The strain-displacement relations follow the von-Karman’s assumptions [31]

(5) $$\varepsilon_{xx}=\frac{\partial u(x,y,t)}{\partial x}-z\frac{\partial^{2}w_{b}(x,y,t)}{\partial x^{2}}-f(z)\frac{\partial^{2}w_{s}(x,y,t)}{\partial x^{2}},\;\varepsilon_{yy}=\frac{\partial v(x,y,t)}{\partial y}-z\frac{\partial^{2}w_{b}(x,y,t)}{\partial y^{2}}-f(z)\frac{\partial^{2}w_{s}(x,y,t)}{\partial y^{2}},\;\varepsilon_{zz}=-\frac{\partial^{2}\left(f(z)\varphi (x,y,t)\right)}{\partial z^{2}},\;\gamma_{xy}=\frac{\partial v(x,y,t)}{\partial x}-2z\frac{\partial^{2}w_{b}(x,y,t)}{\partial x\partial y}-2f(z)\frac{\partial^{2}w_{s}(x,y,t)}{\partial x\partial y}+\frac{\partial u(x,y,t)}{\partial y},\;\gamma_{xz}=\frac{\partial w_{s}(x,y,t)}{\partial x}+(1-f^{\prime }(z))\frac{\partial \varphi (x,y,t)}{\partial x}-f^{\prime }(z)\frac{\partial w_{s}(x,y,t)}{\partial x},\;\gamma_{yz}=\frac{\partial w_{s}(x,y,t)}{\partial y}+(1-f^{\prime }(z))\frac{\partial \varphi (x,y,t)}{\partial y}-f^{\prime }(z)\frac{\partial w_{s}(x,y,t)}{\partial y},$$

whereas the constitutive equations for the honeycomb core and FG-GPLs face sheets, read as follows [33]

(6) $${\left\{\begin{array}{c} \sigma_{xx}\\ \begin{array}{l} \sigma_{yy}\\ \sigma_{zz} \end{array}\\ \sigma_{xy}\\ \sigma_{yz}\\ \sigma_{xz} \end{array}\right\}}^{c,f}=\left[\begin{array}{cccccc} C_{11} & C_{12} & C_{13} & 0 & 0 & 0\\ C_{12} & C_{22} & C_{23} & 0 & 0 & 0\\ C_{13} & C_{23} & C_{33} & 0 & 0 & 0\\ 0 & 0 & 0 & C_{44} & 0 & 0\\ 0 & 0 & 0 & 0 & C_{55} & 0\\ 0 & 0 & 0 & 0 & 0 & C_{66} \end{array}\right]\left\{\begin{array}{c} \varepsilon_{xx}\\ \begin{array}{l} \varepsilon_{yy}\\ \varepsilon_{zz} \end{array}\\ \gamma_{xy}\\ \gamma_{yz}\\ \gamma_{xz} \end{array}\right\}$$

where *C_ij_* are the elastic constants for each part of the sandwich structure. More specifically, for the honeycomb core, the elastic constants read as follows [29]

(7) $$\begin{array}{l} C_{11c}=\frac{E_{11}(-\nu_{23}\nu_{32}+1)}{\delta },\;C_{22c}=\frac{E_{22}(-\nu_{13}\nu_{31}+1)}{\delta },\\ C_{33c}=\frac{E_{33}(-\nu_{12}\nu_{21}+1)}{\delta },\;C_{12c}=C_{21c}=\frac{E_{11}(\nu_{23}\nu_{31}+\nu_{21})}{\delta },\\ C_{13c}=C_{31c}=\frac{E_{11}(\nu_{21}\nu_{32}+\nu_{31})}{\delta },\;C_{23c}=C_{32c}=\frac{E_{22}(\nu_{12}\nu_{31}+\nu_{32})}{\delta },\\ C_{44c}=G_{23},\;C_{55c}=G_{13},\;C_{66c}=G_{12} \end{array}$$

where

(8) $$\delta =1-2\nu_{12}\nu_{13}\nu_{32}-\nu_{12}\nu_{21}-\nu_{13}\nu_{31}-\nu_{23}\nu_{32}$$

and

(9) $$E_{11}=E_{h}\frac{\cos\theta_{0}(1-\gamma_{0}^{2}\cot^{2}\theta_{0})}{\sin^{2}\theta_{0}(\phi_{0}+\sin\theta_{0})}\gamma_{0}^{3},$$

(10) $$E_{22}=E_{h}\frac{\left(1-\gamma_{0}^{2}(\phi_{0}\sec^{2}\theta_{0}+\tan^{2}\theta_{0}))(\phi_{0}+\sin\theta_{0}\right)}{\cos^{3}\theta_{0}}\gamma_{0}^{3},$$

(11) $$E_{33}=E_{h}\frac{2+\phi_{0}}{2\cos\theta_{0}(\phi_{0}+\sin\theta_{0})}\gamma_{0},$$

(12) $$G_{12}=E_{h}\frac{\left(\phi_{0}+\sin\theta_{0}\right)}{\phi_{0}^{2}(1+2\phi_{0})\cos\theta_{0}}\gamma_{0}^{3},$$

(13) $$G_{13}=G_{h}\frac{\cos\theta_{0}}{\left(\phi_{0}+\sin\theta_{0}\right)}\gamma_{0},$$

(14) $$G_{23}=G_{h}\left(\frac{\left(\phi_{0}+\sin\theta_{0}\right)}{(1+2\phi_{0})\cos\theta_{0}}+\frac{\left(\phi_{0}+2\sin^{2}\theta_{0}\right)}{2(\phi_{0}+\sin\theta_{0})}\right)\frac{\gamma_{0}}{2\cos\theta_{0}},$$

(15) $$\rho^{c}=\rho_{h}\frac{2+\phi_{0}}{2\cos\theta_{0}(\phi_{0}+\sin\theta_{0})}\gamma_{0},$$

(16) $$\nu_{12}=\frac{\cos^{2}\theta_{0}(1-\gamma_{0}^{2}\csc^{2}\theta_{0})}{\sin\theta_{0}(\phi_{0}+\sin\theta_{0})},$$

(17) $$\nu_{21}=\frac{(1+\phi_{0})(1-\gamma_{0}^{2}\sec^{2}\theta_{0})\cos^{2}\theta_{0}}{\sin\theta_{0}(\phi_{0}+\sin\theta_{0})},$$

(18) $$\nu_{31}=\nu_{32}=\nu_{h}$$

In the relations above, the Young’s modulus, shear modulus, density, and Poisson’s ratio are defined in a homogenized form by means of the mechanical properties *E_h_*, *G_h_*, *ρ_h_* and *v_h_* of the honeycomb material [34]. Besides, *ϕ*_0_ is the internal cells angle of the honeycomb structure; *φ*_0_ = *h*_0_/*l*_0_ and *γ*_0_ = *t*_0_/*l*_0_ stand for the internal aspect ratio and dimensionless cells thickness, respectively, in which *h*_0_, *l*_0_ and *t*_0_ are the geometrical parameters defining the hexagonal cells as represented in Figure 1. For FG-GPLs reinforced face sheets, the elastic constants *C_ijf_* are given by

(19) $$C_{11f}=C_{22f}=C_{33f}=\frac{\left(1-\nu (z))E(z\right)}{\left(1+\nu (z))(1-2\nu (z)\right)},\;C_{12f}=C_{13f}=C_{23f}=\frac{\nu (z)E(z)}{\left(1+\nu (z))(1-2\nu (z)\right)},\;C_{44f}=C_{55f}=C_{66f}=\frac{E(z)}{2(1+\nu (z))}$$

The mechanical properties for both face sheets vary throughout the thickness, and they are clearly function of the effective material properties, defined, in turn, by means of the Halpin-Tsai micromechanical model, as follows [35]

(20) $$E(z)=\frac{3}{8}\frac{1+\zeta_{L}\eta_{L}V_{GPL}}{1-\eta_{L}V_{GPL}}E_{M}+\frac{5}{8}\frac{1+\zeta_{W}\eta_{W}V_{GPL}}{1-\eta_{W}V_{GPL}}E_{M}$$

In the last relation, *E_M_* denotes the Young’s modulus of the matrix; *V_GPL_* refers to the volume fraction of GPLs; *ζ_L_, ζ_W_, η_L_* and *η_W_* are the geometrical properties of GPLs, i.e.,

(21) $$\begin{array}{l} \zeta_{L}=2\frac{L_{GPL}}{h_{GPL}},\;\eta_{W}=\left(E_{GPL}/E_{M}-1\right)/\left(E_{GPL}/E_{M}+\zeta_{W}\right),\\ \zeta_{W}=2\frac{w_{GPL}}{h_{GPL}},\;\eta_{L}=\left(E_{GPL}/E_{M}-1\right)/\left(E_{GPL}/E_{M}+\zeta_{L}\right), \end{array}$$

*L_GPL_, h_GPL_*, *w_GPL_* and *E_GPL_* being the length, thickness, width and Young’s modulus of GPLs, respectively. It is noteworthy that the summation of GPLs and matrix volume fractions equals one, where the GPLs volume fraction is determined as

(22) VGPL=gGPL(z)gGPL(z)+(ρGPLρM)(1−gGPL(z))

where *ρ_GPL_* and *ρ_M_* refer to the density of the reinforcement phase and matrix, respectively. Moreover, *g_GPL_* is the weight fraction of GPLs that obey the following relations for three different dispersion patterns though the face sheets thicknesses [36]

For a parabolic pattern

(23) $$g_{GPL}(z)=\frac{4}{h_{f}^{2}}\lambda_{P}W_{GPL}z^{2}$$

For a linear pattern

(24) $$g_{GPL}(z)=\lambda_{L}W_{GPL}(\frac{1}{2}\pm \frac{z}{h_{f}})$$

in which the positive and negative signs are related to the top and bottom face sheets, respectively. For a uniform pattern

(25) $$g_{GPL}(z)=\lambda_{U}W_{GPL}$$

In Equations (23)–(25), *λ*_P_, λ_L_ and λ_U_ are the gradient index of GPLs for their parabolic, linear, and uniform dispersion patterns, referred to the total GPLs content, as reported in Table 1.

The further properties for the face sheets are the Poisson’s ratio and density, which are determined via the ERM as [37]

(26) $$\rho (z)=\rho_{GPL}V_{GPL}+\rho_{M}V_{M},$$

(27) $$\nu (z)=\nu_{GPL}V_{GPL}+\nu_{M}V_{M}$$

## 3. Governing Equations of the Problem

The Hamilton’s principle is here applied to gain the governing equations of the problem [38]

(28) $$\int_{t_{1}}^{t_{2}}\delta \left(\Lambda -K-\Pi \right)dt=0$$

where *Π*, *Λ* and *K* denote the applied external work, the strain energy, and the kinetic energy for the sandwich structure, respectively. The strain energy of the system consists of two parts: the classical strain energy and the energy component from the MCST. The following relation is used to define the total strain energy for the selected sandwich structure [39]

(29) $$\Lambda =\frac{1}{2}\left(\begin{array}{l} \int_{x}\int_{y}\int_{core}\left(\sigma_{ij}^{c}\varepsilon_{ij}+m_{ij}^{c}\chi_{ij}\right)\;dz\;dy\;dx\;\\ \;\;\;\;\;\;\;+\int_{x}\int_{y}\int_{faces}\left(\sigma_{ij}^{f}\varepsilon_{ij}+m_{ij}^{f}\chi_{ij}\right)\;dz\;dy\;dx\; \end{array}\right);\;i,j=x,y,z$$

where *m_ij_* and *χ_ij_* stand for the higher-order stresses and symmetric rotation gradient tensor, respectively, defined in the following

(30) $$m_{ij}=2l_{m}^{2}\mu \chi_{ij};\;(i,j=x,y,z)\;$$

where *l_m_* is the MCST material length scale parameter, and *μ* is the Lame’s parameter. Moreover, the components of the symmetric rotation gradient tensor can be determined using the following compact relation

(31) $$\chi_{ij}=\frac{1}{2}\left(\Theta_{i,j}+\Theta_{j,i}\right)$$

This means that

(32) $$\begin{array}{lll} \chi_{xx}=\frac{\partial }{\partial x}\Theta_{x}, & \chi_{yy}=\frac{\partial }{\partial y}\Theta_{y}, & \chi_{zz}=\frac{\partial }{\partial z}\Theta_{z},\\ \chi_{xy}=\frac{1}{2}\left(\frac{\partial }{\partial y}\Theta_{x}+\frac{\partial }{\partial x}\Theta_{y}\right), & \;\chi_{yz}=\frac{1}{2}\left(\frac{\partial }{\partial z}\Theta_{y}+\frac{\partial }{\partial y}\Theta_{z}\right), & \chi_{xz}=\frac{1}{2}(\frac{\partial }{\partial z}\Theta_{x}+\frac{\partial }{\partial x}\Theta_{z}) \end{array}$$

in which, the infinitesimal rotation vector *Θ* is defined as

(33) $$\Theta_{i}=\frac{1}{2}{\left(curl(u)\right)}_{,i}$$

which means

(34) $$\Theta_{x}=\frac{1}{2}\left(\frac{\partial }{\partial y}W(x,y,z,t)-\frac{\partial }{\partial z}V(x,y,z,t)\right),\;\Theta_{y}=\frac{1}{2}\left(\frac{\partial }{\partial z}U(x,y,z,t)-\frac{\partial }{\partial x}W(x,y,z,t)\right),\;\Theta_{z}=\frac{1}{2}\left(\frac{\partial }{\partial x}V(x,y,z,t)-\frac{\partial }{\partial y}U(x,y,z,t)\right)$$

In addition, the kinetic energy for the whole microstructure can be defined as [40].

(35) $$K=\frac{1}{2}\underset{x}{\int }\underset{y}{\int }\overset{+h/2}{\underset{-h/2}{\int }}\rho_{c,f}\left(z\right)\left[{\left(\frac{\partial U}{\partial t}\right)}^{2}+{\left(\frac{\partial V}{\partial t}\right)}^{2}+\;{\left(\frac{\partial W}{\partial t}\right)}^{2}\right]dzdydx$$

where *U, V* and *W* refer to the displacement components introduced in Equation (1).

For a structure resting on a visco-Pasternak elastic foundation, the external work due to the substrate can be defined as follows [41]

(36) $$\Pi =\underset{x}{\int }\underset{y}{\int }\frac{1}{2}\left(\begin{array}{c} K_{W}\text{​}{\left(w_{b}+w_{s}\right)}^{2}-K_{G}\left(w_{b}+w_{s}\right)\text{​}\frac{\partial^{2}\left(w_{b}+w_{s}\right)}{\partial x^{2}}-\\ K_{G}\left(w_{b}+w_{s}\right)\frac{\partial^{2}\left(w_{b}+w_{s}\right)}{\partial y^{2}}+\\ C_{d}\left(w_{b}+w_{s}\right)\text{​}\frac{\partial \left(w_{b}+w_{s}\right)}{\partial t} \end{array}\right)dxdy\text{​}$$

where *K_W_* is the Winkler parameter, *K_G_* is the shear layer parameter, and *C_d_* denotes the damping parameter, respectively. By substitution of Equations (29), (35), (36) into the Hamilton’s principle (28), after a mathematical manipulation we get the following governing equations of motion in terms of displacement field

δu:

(37) $$\begin{array}{l} -C_{110}\frac{\partial^{2}u(x,y,t)}{\partial x^{2}}+C_{111}\frac{\partial^{3}w_{b}(x,y,t)}{\partial x^{3}}+F_{110}\frac{\partial^{3}w_{s}(x,y,t)}{\partial x^{3}}+\\ +C_{121}\frac{\partial^{3}w_{b}(x,y,t)}{\partial x\partial y^{2}}+F_{120}\frac{\partial^{3}w_{s}(x,y,t)}{\partial x\partial y^{2}}-E_{130}\frac{\partial \varphi (x,y,t)}{\partial x}+\\ -C_{440}\frac{\partial^{2}v(x,y,t)}{\partial x\partial y}-\frac{1}{4}K\frac{\partial^{4}v(x,y,t)}{\partial x\partial y^{3}}-C_{120}\frac{\partial^{2}v(x,y,t)}{\partial x\partial y}+\\ +2C_{441}\frac{\partial^{3}w_{b}(x,y,t)}{\partial x\partial y^{2}}+2F_{440}\frac{\partial^{3}w_{s}(x,y,t)}{\partial x\partial y^{2}}-C_{440}\frac{\partial^{2}u(x,y,t)}{\partial y^{2}}+\\ +\frac{1}{4}K\frac{\partial^{4}u(x,y,t)}{\partial y^{4}}-\frac{1}{4}K\frac{\partial^{4}v(x,y,t)}{\partial x^{3}\partial y}+\frac{1}{4}K\frac{\partial^{4}u(x,y,t)}{\partial x^{2}\partial y^{2}}+\\ -I_{0}\frac{\partial^{2}u(x,y,t)}{\partial t^{2}}+I_{1}\frac{\partial^{3}w_{b}(x,y,t)}{\partial t^{2}\partial x}+I_{3}\frac{\partial^{3}w_{s}(x,y,t)}{\partial t^{2}\partial x}=0 \end{array}$$

δv:

(38) $$\begin{array}{l} -C_{120}\frac{\partial^{2}u(x,y,t)}{\partial x\partial y}+C_{121}\frac{\partial^{3}w_{b}(x,y,t)}{\partial x^{2}\partial y}+F_{120}\frac{\partial^{3}w_{s}(x,y,t)}{\partial x^{2}\partial y}-C_{220}\frac{\partial^{2}v(x,y,t)}{\partial y^{2}}+\\ +C_{221}\frac{\partial^{3}w_{b}(x,y,t)}{\partial y^{3}}+F_{220}\frac{\partial^{3}w_{s}(x,y,t)}{\partial y^{3}}-E_{230}\frac{\partial \varphi (x,y,t)}{\partial y}-C_{440}\frac{\partial^{2}v(x,y,t)}{\partial x^{2}}+\\ +2C_{441}\frac{\partial^{3}w_{b}(x,y,t)}{\partial x^{2}\partial y}+2F_{440}\frac{\partial^{3}w_{s}(x,y,t)}{\partial x^{2}\partial y}-C_{440}\frac{\partial^{2}u(x,y,t)}{\partial y\partial x}+\frac{1}{4}K\frac{\partial^{4}v(x,y,t)}{\partial x^{4}}+\\ -\frac{1}{4}K\frac{\partial^{4}u(x,y,t)}{\partial x^{3}\partial y}+\frac{1}{4}K\frac{\partial^{4}v(x,y,t)}{\partial x^{2}\partial y^{2}}-\frac{1}{4}K\frac{\partial^{4}u(x,y,t)}{\partial x\partial y^{3}}+\\ -I_{0}\frac{\partial^{2}v(x,y,t)}{\partial t^{2}}+I_{1}\frac{\partial^{3}w_{b}(x,y,t)}{\partial t^{2}\partial y}+I_{3}\frac{\partial^{3}w_{s}(x,y,t)}{\partial t^{2}\partial y}=0 \end{array}$$

$$\delta w_{b}:$$

(39) $$\begin{array}{l} -C_{121}\frac{\partial^{3}u(x,y,t)}{\partial x\partial y^{2}}+C_{122}\frac{\partial^{4}w_{b}(x,y,t)}{\partial x^{2}\partial y^{2}}+F_{121}\frac{\partial^{4}w_{s}(x,y,t)}{\partial x^{2}\partial y^{2}}-C_{221}\frac{\partial^{3}v(x,y,t)}{\partial y^{3}}+\\ +C_{222}\frac{\partial^{4}w_{b}(x,y,t)}{\partial y^{4}}+F_{221}\frac{\partial^{4}w_{s}(x,y,t)}{\partial y^{4}}-E_{231}\frac{\partial^{2}\varphi (x,y,t)}{\partial y^{2}}-2C_{441}\frac{\partial^{3}v(x,y,t)}{\partial x^{2}\partial y}+\\ +4C_{442}\frac{\partial^{4}w_{b}(x,y,t)}{\partial x^{2}\partial y^{2}}+4F_{441}\frac{\partial^{4}w_{s}(x,y,t)}{\partial x^{2}\partial y^{2}}-2C_{441}\frac{\partial^{3}u(x,y,t)}{\partial y^{2}\partial x}+\\ -C_{111}\frac{\partial^{3}u(x,y,t)}{\partial x^{3}}+C_{112}\frac{\partial^{4}w_{b}(x,y,t)}{\partial x^{4}}+F_{111}\frac{\partial^{4}w_{s}(x,y,t)}{\partial x^{4}}-C_{121}\frac{\partial^{3}v(x,y,t)}{\partial x^{2}\partial y}+\\ +C_{122}\frac{\partial^{4}w_{b}(x,y,t)}{\partial x^{2}\partial y^{2}}+2K\frac{\partial^{4}w_{b}(x,y,t)}{\partial x^{2}\partial y^{2}}+K\frac{\partial^{4}w_{s}(x,y,t)}{\partial x^{2}\partial y^{2}}+\\ +K_{0}\frac{\partial^{4}\varphi (x,y,t)}{\partial x^{2}\partial y^{2}}-K_{1}\frac{\partial^{4}w_{s}(x,y,t)}{\partial x^{2}\partial y^{2}}+K\frac{\partial^{4}w_{b}(x,y,t)}{\partial y^{4}}+\frac{1}{2}K\frac{\partial^{4}w_{s}(x,y,t)}{\partial y^{4}}+\\ +\frac{1}{2}K_{0}\frac{\partial^{4}\varphi (x,y,t)}{\partial y^{4}}+\frac{1}{2}K_{1}\frac{\partial^{4}w_{s}(x,y,t)}{\partial y^{4}}+K\frac{\partial^{4}w_{b}(x,y,t)}{\partial y^{4}}+\frac{1}{2}K_{1}\frac{\partial^{4}w_{s}(x,y,t)}{\partial x^{4}}+\\ +\frac{1}{2}K\frac{\partial^{4}w_{s}(x,y,t)}{\partial x^{4}}+\frac{1}{2}K_{0}\frac{\partial^{4}\varphi (x,y,t)}{\partial x^{4}}+F_{121}\frac{\partial^{4}w_{s}(x,y,t)}{\partial x^{2}\partial y^{2}}-E_{131}\frac{\partial^{4}\varphi (x,y,t)}{\partial x^{2}}+\\ -C_{d}\frac{\partial w_{b}(x,y,t)}{\partial t}-C_{d}\frac{\partial w_{s}(x,y,t)}{\partial t}+K_{G}\frac{\partial^{2}w_{b}(x,y,t)}{\partial x^{2}}+K_{G}\frac{\partial^{2}w_{s}(x,y,t)}{\partial x^{2}}+\\ +K_{G}g\frac{\partial^{2}\varphi (x,y,t)}{\partial x^{2}}+K_{G}\frac{\partial^{2}w_{b}(x,y,t)}{\partial y^{2}}+K_{G}\frac{\partial^{2}w_{s}(x,y,t)}{\partial y^{2}}+K_{G}g\frac{\partial^{2}\varphi (x,y,t)}{\partial y^{2}}+\\ -C_{d}g\frac{\partial \varphi (x,y,t)}{\partial t}-K_{W}g\varphi (x,y,t)-K_{W}w_{s}(x,y,t)-K_{W}w_{b}(x,y,t)-I_{1}\frac{\partial^{3}u(x,y,t)}{\partial t^{2}\partial x}+\\ +I_{2}\frac{\partial^{4}w_{b}(x,y,t)}{\partial t^{2}\partial x^{2}}+I_{5}\frac{\partial^{4}w_{s}(x,y,t)}{\partial t^{2}\partial x^{2}}-I_{1}\frac{\partial^{3}v(x,y,t)}{\partial t^{2}\partial y}+I_{2}\frac{\partial^{4}w_{b}(x,y,t)}{\partial t^{2}\partial y^{2}}+\\ +I_{5}\frac{\partial^{4}w_{s}(x,y,t)}{\partial t^{2}\partial y^{2}}-I_{0}\frac{\partial^{2}w_{b}(x,y,t)}{\partial t^{2}}-I_{0}\frac{\partial^{2}w_{s}(x,y,t)}{\partial t^{2}}-I_{4}\frac{\partial^{2}\varphi (x,y,t)}{\partial t^{2}}=0 \end{array}$$

$$\delta w_{s}:$$

(40) $$\begin{array}{l} \frac{1}{2}K_{1}\frac{\partial^{4}w_{b}(x,y,t)}{\partial x^{4}}+\frac{1}{4}K_{3}\frac{\partial^{4}w_{s}(x,y,t)}{\partial x^{4}}+\frac{1}{4}K_{2}\frac{\partial^{4}\varphi (x,y,t)}{\partial x^{4}}-F_{111}\frac{\partial^{4}w_{b}(x,y,t)}{\partial x^{4}}+\\ +\frac{1}{2}K_{1}\frac{\partial^{4}w_{b}(x,y,t)}{\partial y^{4}}+\frac{1}{4}K_{3}\frac{\partial^{4}w_{s}(x,y,t)}{\partial x^{4}}+\frac{1}{4}K_{2}\frac{\partial^{4}\varphi (x,y,t)}{\partial x^{4}}-\frac{1}{4}K_{5}\frac{\partial^{2}w_{s}(x,y,t)}{\partial x^{2}}+\\ -\frac{1}{4}K_{5}\frac{\partial^{2}w_{s}(x,y,t)}{\partial y^{2}}-\frac{1}{4}K_{4}\frac{\partial^{2}\varphi (x,y,t)}{\partial x^{2}}-\frac{1}{4}K_{4}\frac{\partial^{2}\varphi (x,y,t)}{\partial y^{2}}-F_{110}\frac{\partial^{3}u(x,y,t)}{\partial x^{3}}+\\ -F_{120}\frac{\partial^{3}v(x,y,t)}{\partial x^{2}\partial y}+F_{121}\frac{\partial^{4}w_{b}(x,y,t)}{\partial y^{2}\partial x^{2}}+F_{122}\frac{\partial^{4}w_{s}(x,y,t)}{\partial y^{2}\partial x^{2}}-E_{132}\frac{\partial^{2}\varphi (x,y,t)}{\partial x^{2}}+\\ -F_{120}\frac{\partial^{3}u(x,y,t)}{\partial y^{2}\partial x}+F_{121}\frac{\partial^{4}w_{b}(x,y,t)}{\partial x^{2}\partial y^{2}}+F_{112}\frac{\partial^{4}w_{s}(x,y,t)}{\partial x^{4}}+F_{122}\frac{\partial^{4}w_{s}(x,y,t)}{\partial x^{2}\partial y^{2}}+\\ -F_{220}\frac{\partial^{3}v(x,y,t)}{\partial y^{3}}+F_{221}\frac{\partial^{4}w_{b}(x,y,t)}{\partial y^{4}}+F_{222}\frac{\partial^{4}w_{s}(x,y,t)}{\partial y^{4}}-E_{232}\frac{\partial^{2}\varphi (x,y,t)}{\partial y^{2}}+\\ -G_{550}\frac{\partial^{2}w_{s}(x,y,t)}{\partial x^{2}}-G_{550}\frac{\partial^{2}\varphi (x,y,t)}{\partial x^{2}}+G_{551}\frac{\partial^{2}w_{s}(x,y,t)}{\partial x^{2}}+G_{551}\frac{\partial^{2}\varphi (x,y,t)}{\partial x^{2}}+\\ +G_{661}\frac{\partial^{2}w_{s}(x,y,t)}{\partial y^{2}}+G_{661}\frac{\partial^{2}\varphi (x,y,t)}{\partial y^{2}}+K_{1}\frac{\partial^{4}w_{b}(x,y,t)}{\partial x^{2}\partial y^{2}}+\frac{1}{2}K_{2}\frac{\partial^{4}\varphi (x,y,t)}{\partial x^{2}\partial y^{2}}+\\ +\frac{1}{2}K_{3}\frac{\partial^{4}w_{s}(x,y,t)}{\partial x^{2}\partial y^{2}}+\frac{1}{2}K_{1}\frac{\partial^{4}w_{s}(x,y,t)}{\partial y^{4}}+\frac{1}{2}K\frac{\partial^{4}w_{b}(x,y,t)}{\partial x^{4}}+\frac{1}{2}K_{1}\frac{\partial^{4}w_{s}(x,y,t)}{\partial x^{4}}+\\ +\frac{1}{4}K\frac{\partial^{4}w_{s}(x,y,t)}{\partial x^{4}}+\frac{1}{4}K_{0}\frac{\partial^{4}\varphi (x,y,t)}{\partial x^{4}}+K\frac{\partial^{4}w_{b}(x,y,t)}{\partial x^{2}\partial y^{2}}+\frac{1}{2}K\frac{\partial^{4}w_{s}(x,y,t)}{\partial x^{2}\partial y^{2}}+\\ +K_{1}\frac{\partial^{4}w_{s}(x,y,t)}{\partial x^{2}\partial y^{2}}+\frac{1}{2}K_{0}\frac{\partial^{4}\varphi (x,y,t)}{\partial x^{2}\partial y^{2}}+\frac{1}{2}K\frac{\partial^{4}w_{b}(x,y,t)}{\partial y^{4}}+\frac{1}{4}K\frac{\partial^{4}w_{s}(x,y,t)}{\partial y^{4}}+\\ \frac{1}{4}K_{0}\frac{\partial^{4}\varphi (x,y,t)}{\partial y^{4}}-2F_{440}\frac{\partial^{3}v(x,y,t)}{\partial x^{2}\partial y}+4F_{441}\frac{\partial^{4}w_{b}(x,y,t)}{\partial x^{2}\partial y^{2}}+4F_{442}\frac{\partial^{4}w_{s}(x,y,t)}{\partial x^{2}\partial y^{2}}+\\ -2F_{440}\frac{\partial^{3}u(x,y,t)}{\partial x\partial y^{2}}-C_{d}\frac{\partial w_{b}(x,y,t)}{\partial t}-C_{d}\frac{\partial w_{s}(x,y,t)}{\partial t}-C_{d}g\frac{\partial \varphi (x,y,t)}{\partial t}+\\ +K_{G}\frac{\partial^{2}w_{s}(x,y,t)}{\partial x^{2}}+K_{G}\frac{\partial^{2}w_{b}(x,y,t)}{\partial x^{2}}+K_{G}g\frac{\partial^{2}\varphi (x,y,t)}{\partial x^{2}}+K_{G}\frac{\partial^{2}w_{s}(x,y,t)}{\partial y^{2}}+\\ +K_{G}\frac{\partial^{2}w_{b}(x,y,t)}{\partial y^{2}}+K_{G}g\frac{\partial^{2}\varphi (x,y,t)}{\partial y^{2}}-K_{W}g\varphi (x,y,t)-K_{W}w_{s}(x,y,t)-K_{W}w_{b}(x,y,t)+\\ -I_{3}\frac{\partial^{3}u(x,y,t)}{\partial t^{2}\partial x}+I_{5}\frac{\partial^{4}w_{b}(x,y,t)}{\partial t^{2}\partial x^{2}}+I_{6}\frac{\partial^{4}w_{s}(x,y,t)}{\partial t^{2}\partial x^{2}}-I_{3}\frac{\partial^{3}v(x,y,t)}{\partial t^{2}\partial y}+\\ +I_{5}\frac{\partial^{4}w_{b}(x,y,t)}{\partial t^{2}\partial y^{2}}+I_{6}\frac{\partial^{4}w_{s}(x,y,t)}{\partial t^{2}\partial y^{2}}-I_{0}\frac{\partial^{2}w_{b}(x,y,t)}{\partial t^{2}}-I_{0}\frac{\partial^{2}w_{s}(x,y,t)}{\partial t^{2}}+\\ -I_{4}\frac{\partial^{2}\varphi (x,y,t)}{\partial t^{2}}=0 \end{array}$$

δφ:

(41) $$\begin{array}{l} -\frac{1}{2}K_{1}\frac{\partial^{4}w_{b}(x,y,t)}{\partial y^{4}}-\frac{1}{4}K_{2}\frac{\partial^{4}\varphi (x,y,t)}{\partial y^{4}}-\frac{1}{4}K_{3}\frac{\partial^{4}w_{s}(x,y,t)}{\partial y^{4}}-\frac{1}{2}K_{1}\frac{\partial^{4}w_{b}(x,y,t)}{\partial x^{4}}+\\ +\frac{1}{4}K_{3}\frac{\partial^{4}w_{s}(x,y,t)}{\partial x^{4}}+\frac{1}{4}K_{2}\frac{\partial^{4}\varphi (x,y,t)}{\partial x^{4}}+\frac{1}{4}K_{4}\frac{\partial^{2}\varphi (x,y,t)}{\partial y^{2}}+\frac{1}{4}K_{5}\frac{\partial^{2}w_{s}(x,y,t)}{\partial y^{2}}+\\ +\frac{1}{4}K_{5}\frac{\partial^{2}w_{s}(x,y,t)}{\partial x^{2}}+\frac{1}{4}K_{4}\frac{\partial^{2}\varphi (x,y,t)}{\partial x^{2}}-G_{550}\frac{\partial^{2}w_{s}(x,y,t)}{\partial x^{2}}-G_{550}\frac{\partial^{2}\varphi (x,y,t)}{\partial x^{2}}+\\ +G_{551}\frac{\partial^{2}w_{s}(x,y,t)}{\partial x^{2}}+G_{551}\frac{\partial^{2}\varphi (x,y,t)}{\partial x^{2}}+G_{661}\frac{\partial^{2}w_{s}(x,y,t)}{\partial y^{2}}+G_{661}\frac{\partial^{2}\varphi (x,y,t)}{\partial y^{2}}+\\ -K_{1}\frac{\partial^{4}w_{b}(x,y,t)}{\partial x^{2}\partial y^{2}}-\frac{1}{2}K_{2}\frac{\partial^{4}\varphi (x,y,t)}{\partial y^{2}\partial x^{2}}-\frac{1}{2}K_{3}\frac{\partial^{4}w_{s}(x,y,t)}{\partial y^{2}\partial x^{2}}+\\ +\frac{1}{4}K_{0}\frac{\partial^{4}\varphi (x,y,t)}{\partial x^{4}}-\frac{1}{4}K\frac{\partial^{4}w_{s}(x,y,t)}{\partial x^{4}}-\frac{1}{2}K\frac{\partial^{4}w_{b}(x,y,t)}{\partial x^{4}}+\frac{1}{4}K_{0}\frac{\partial^{4}\varphi (x,y,t)}{\partial y^{4}}+\\ +K\frac{\partial^{4}w_{b}(x,y,t)}{\partial y^{2}\partial x^{2}}+\frac{1}{2}K\frac{\partial^{4}w_{s}(x,y,t)}{\partial y^{2}\partial x^{2}}+\frac{1}{2}K_{0}\frac{\partial^{4}\varphi (x,y,t)}{\partial y^{2}\partial x^{2}}+\frac{1}{4}K\frac{\partial^{4}w_{b}(x,y,t)}{\partial y^{4}}+\\ +\frac{1}{4}K\frac{\partial^{4}w_{s}(x,y,t)}{\partial y^{4}}+E_{330}\varphi (x,y,t)+E_{230}\frac{\partial v(x,y,t)}{\partial y}-E_{131}\frac{\partial^{2}w_{b}(x,y,t)}{\partial x^{2}}-E_{132}\frac{\partial^{2}w_{s}(x,y,t)}{\partial x^{2}}+\\ -E_{232}\frac{\partial^{2}w_{s}(x,y,t)}{\partial y^{2}}-E_{231}\frac{\partial^{2}w_{b}(x,y,t)}{\partial y^{2}}-G_{660}\frac{\partial^{2}\varphi (x,y,t)}{\partial y^{2}}-G_{660}\frac{\partial^{2}w_{s}(x,y,t)}{\partial y^{2}}+\\ -C_{d}\frac{\partial w_{b}(x,y,t)}{\partial t}-C_{d}\frac{\partial w_{s}(x,y,t)}{\partial t}-C_{d}g^{2}\frac{\partial \varphi (x,y,t)}{\partial t}-K_{W}gw_{b}(x,y,t)-K_{W}gw_{s}(x,y,t)+\\ -K_{W}g^{2}\varphi (x,y,t)+K_{G}g\frac{\partial^{2}w_{s}(x,y,t)}{\partial x^{2}}+K_{G}g\frac{\partial^{2}w_{b}(x,y,t)}{\partial x^{2}}+K_{G}g^{2}\frac{\partial^{2}\varphi (x,y,t)}{\partial x^{2}}+\\ +K_{G}g\frac{\partial^{2}w_{s}(x,y,t)}{\partial y^{2}}+K_{G}g\frac{\partial^{2}w_{b}(x,y,t)}{\partial y^{2}}+K_{G}g^{2}\frac{\partial^{2}\varphi (x,y,t)}{\partial y^{2}}+\\ -I_{4}\frac{\partial^{2}w_{b}(x,y,t)}{\partial t^{2}}+I_{4}\frac{\partial^{2}w_{s}(x,y,t)}{\partial t^{2}}+I_{4}\frac{\partial^{2}\varphi (x,y,t)}{\partial t^{2}}=0 \end{array}$$

More details about the coefficients in Equations (37)–(41), are reported in the Appendix.

## 4. Analytical Solution Procedure

The differential equations of the Equations (37)–(41) are solved analytically according to the Navier’s procedure in this section. Therefore, for a simply supported structure, we consider the following theoretical expressions for the displacement components [42]

(42) $$u\left(x,y,t\right)=U\cos\left(\alpha x\right)\sin\left(\beta y\right)e^{i\omega t},\;v\left(x,y,t\right)=V\sin\left(\alpha x\right)\cos\left(\beta y\right)e^{i\omega t},\;w_{b}\left(x,y,t\right)=W_{b}\sin\left(\alpha x\right)\sin\left(\beta y\right)e^{i\omega t},\;w_{s}\left(x,y,t\right)=W_{s}\sin\left(\alpha x\right)\sin\left(\beta y\right)e^{i\omega t},\;\varphi \left(x,y,t\right)=\Phi \sin\left(\alpha x\right)\sin\left(\beta y\right)e^{i\omega t}$$

in which *U, V, W_s_, W_b_* and *Φ* are the unknown coefficients. In addition, *α* and *β* are defined as *mπ/a* and *nπ/b*, respectively, where *m* and *n* are the mode numbers along the length and width direction, respectively. After substituting Equation (42) into Equations (37)–(41), the equations of motion gain the following compact form

(43) $$\left({\left[K\right]}_{5\times 5}+i\omega {\left[C\right]}_{5\times 5}-\omega^{2}{\left[M\right]}_{5\times 5}\right)\left\{d\right\}=0$$

where [*K*], [*C*], and [*M*] refer to the stiffness matrix, damping matrix, and mass matrix, respectively, whereas {*d*} is the displacement vector. The natural frequencies of the structure are then obtained by solving the classical eigenvalue problem (43).

## 5. Numerical Results

In this section we illustrate the numerical results, in terms of vibration response, for a microsandwich plate with a honeycomb core made of Nomex or Glass phenolic, and Epoxy-reinforced GPLs as face sheets. The Nomex has the following properties: *E_s_* = 3.2 *GPa*, *ρ* = 48 *kg*/*m*^3^, and *ν* = 0.4. For the Glass phenolic, the same properties of Ref. [43] are assumed herein. The Epoxy matrix and GPLs reinforcement phase for the face sheets have the following properties [44]

$$\begin{array}{lll} E_{GPL}=1.01\;TPa, & \rho_{GPL}=1062.5\;kg/m^{3}, & \nu_{GPL}=0.186,\\ L_{GPL}=2.5\;\mu m, & w_{GPL}=1.5\;\mu m, & h_{GPL}=1.5\;nm,\\ E_{M}=130\;GPa, & \rho_{M}=8960\;kg/m^{3}, & \nu_{M}=0.34 \end{array}$$

The microplate has a total height equal to 150 μm, 80% of whose total height corresponds to the core, and the rest is equally divided between the two face sheets. The length of the square plate is ten-fold of its thickness. The internal cell angle, aspect ratio, and dimensionless cells thickness are assumed to be *π*/6, 1, and 0.1, respectively. Moreover, the material length-scale parameter is kept as 15 μm according to Ref. [36]. 

To check for the reliability of our formulation, we compare the results for a single-layer FG-GPL-reinforced square microplate with predictions by Thai et al. [45]. The comparative results are summarized in Table 2, in terms of dimensionless natural frequencies defined as $\Omega =\left(\omega a^{2}/h\right)\sqrt{\rho_{M}/E_{M}}$, for various mode numbers, while considering the effect of the aspect ratio (*a*/*h*) and length-scale parameter-to-total thickness (*l_m_*/*h*). Based on Table 2, a very good agreement is observable between the results from our formulation and those ones from Ref. [45], where some negligible differences are related to the different kinematic assumptions, and/or different solution techniques.

In Table 3, we also summarize the natural frequencies for different mode numbers, as computed according to a MCST or a classical elasticity theory (CET), for a varying internal cell angle from 30° up to 60°. Based on results in Table 3, note that the mode number and internal cell angle yield a reverse effect on the natural frequency of the sandwich microplate, whereby an increasing mode number and a decreasing internal cell angle get higher values of the natural frequencies. It seems also that CET-based predictions are always more conservative than those once based on a MCST, in agreement with findings in Refs. [46,47,48,49,50] from the literature.

Another key aspect of the problem can be the sensitivity of the response to various GPLs dispersions in the Epoxy matrix over a wide range of mode numbers, as listed in Table 4. It is worth noticing that the sandwich structure becomes stiffer for an increased quantity of GPLs as reinforcing phase, and the natural frequency enhances dramatically in each mode number. 

It seems also that a linear dispersion of GPLs in the Epoxy matrix with *λ_L_* = 2 is more effective than other types of distribution with *λ_P_ = λ_U_* = 1 for an overall increase in the structural stiffness. This shows that the GPLs dispersion coefficient plays a crucial role, more than the type of GPLs dispersion, for an increase in the natural frequency.

**Figure 2:**
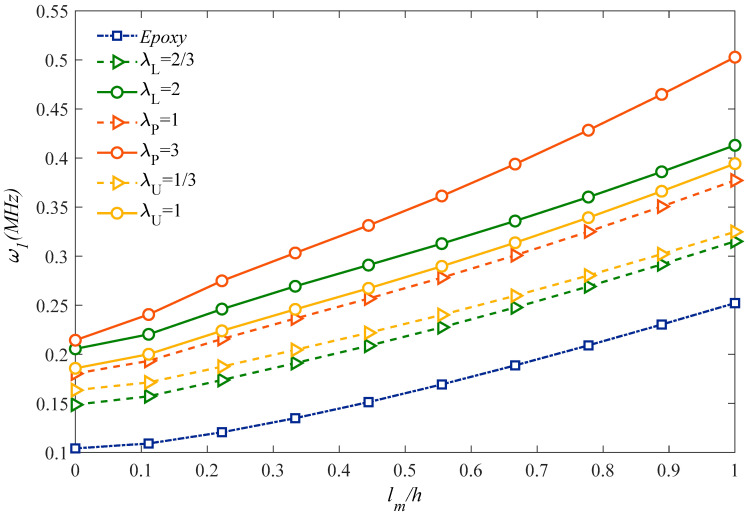
Size and GPLs amount effects on the fundamental natural frequency.

Figure 2 shows the variation of the natural frequency for the sandwich microplate against the *l_m_*/*h* ratio, for different dispersions of GPLs. By increasing *l_m_*/*h* rational value, and keeping constant the total thickness of the sandwich model, the natural frequency increases monotonically, for each fixed value of *λ_L_, λ_P_, λ_U_*. This behavior is due to a reduced flexibility of the sandwich microplate which corresponds to a stiffness and stability enhancement. For each type of GPLs dispersion, a higher distribution coefficient obtains higher natural frequencies.

**Figure 3:**
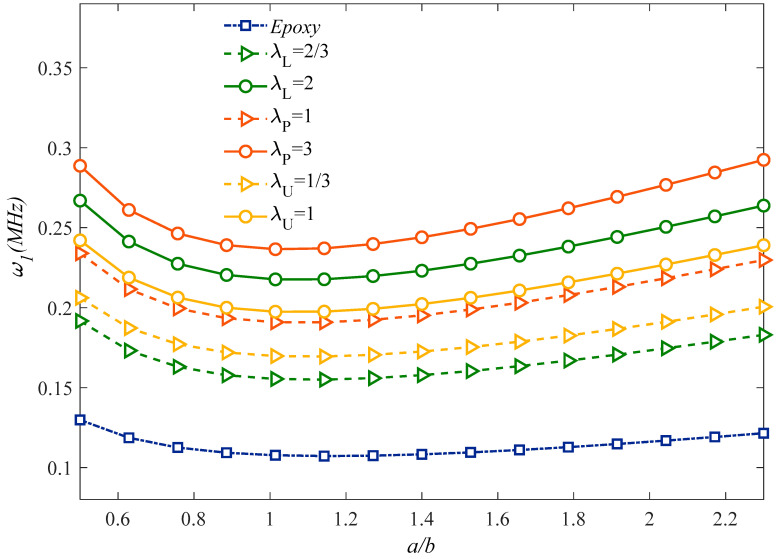
Effect of the aspect ratio and GPLs dispersion pattern on the structural response. (*S* = 225 × 10^−12^).

**Figure 4:**
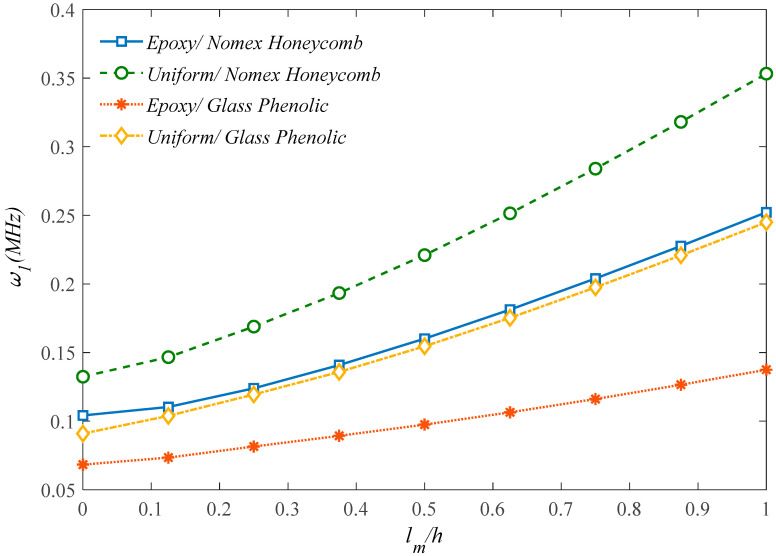
Effect of the core materials on the structural response.

**Figure 5:**
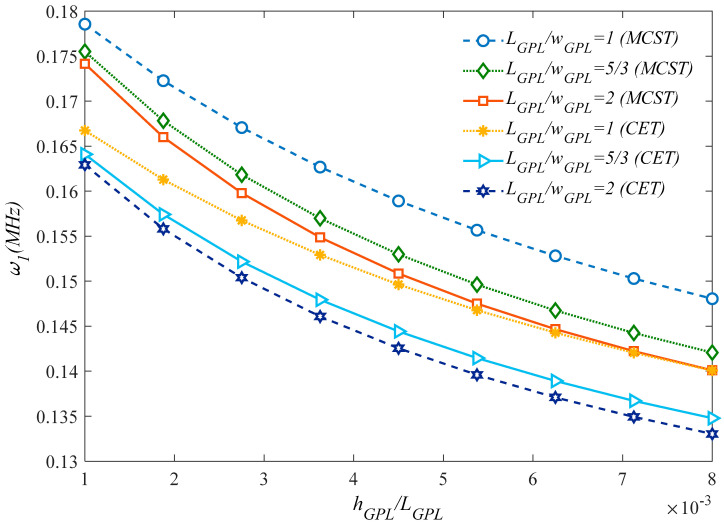
Effect of the GPLs geometry on the structural response, according to the MCST and CET.

Figure 3 plots the effect of the aspect ratio, *a/b*, on the natural frequency of the microstructure for different GPLs dispersion coefficients. For each fixed GPLs dispersion coefficient and type, an increased aspect ratio up to one clearly reduces the natural frequency reaching the minimum value for a cubic sandwich structure. Once this minimum value is passed, the aspect ratio rolling up causes a monotonic increase in the natural frequency for each selected GPLs dispersion coefficient and type. A further systematic analysis is also performed to check for the sensitivity of the natural frequency alternation with the *l_m_*/*h* ratio, under different assumptions for the honeycomb core material in Figure 4. Based the plots in this figure, it is worth observing that the most rigid sandwich microstructure is obtained for a uniform Nomex honeycomb core material, where the most flexible one is reached for an Epoxy/Glass Phenolic core material. All the other results based on an Epoxy/Nomex honeycomb or Uniform/Glass Phenolic core material assumption are very close to each other, and fall always within the previous two cases. As also plotted in Figure 5, the natural frequency decreases monotonically for an increasing geometrical ratio *h_GPL_/L_GPL_* of the reinforcing phase, as predicted by a CET or a MCST, respectively, while assuming three different rational values for *L_GPL_/W_GPL_*, namely, *L_GPL_/W_GPL_* = 1; 5/3; 2. This means that the GPLs length variations (reduction or enhancement) have a direct relationship with the natural frequency, stiffness and rigidity. For each selected theory, an increased value of *L_GPL_/W_GPL_* reduces gradually the natural frequency for each fixed value of *h_GPL_/L_GPL_*. Based on a comparative evaluation of the curves in Figure 5, it can be noted that MCST provides always a higher natural frequency compared to the CET.

**Figure 6:**
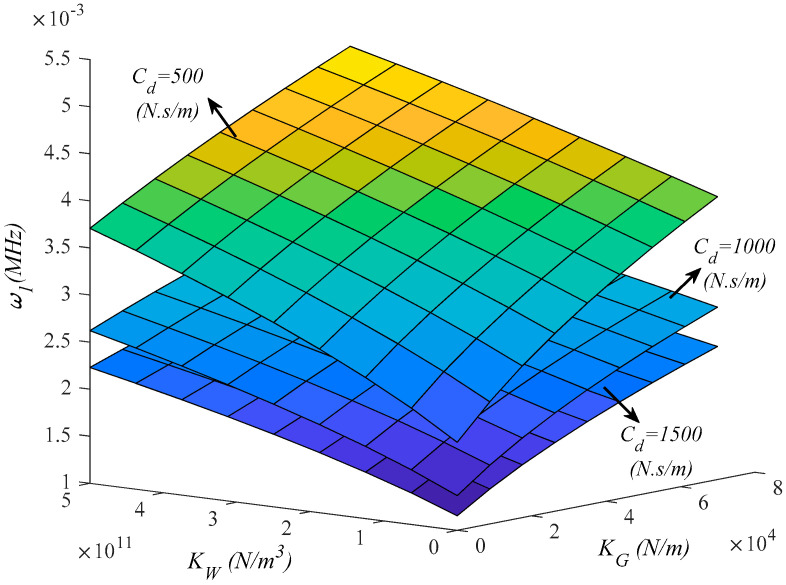
Effect of the viscoelastic foundation parameters on the first natural frequency of the structure (*λ_L_* = 2).

In Figure 6 we analyze the effect of the viscoelastic foundation on the vibration response, while providing the 3D plot of the natural frequency for different combinations of *K_W_*, *K_G_* under three different assumptions for the damping parameter *C_d_* = 500; 1000; 1500 (N·s/m) is provided. By increasing the Winkler and Pasternak parameters (*K_W_*, *K_G_*) the structural stiffness increases together with the natural frequency for each fixed value of *C_d_*. Based on the three plots, it is worth mentioning the great damping effect on the frequency response, where a decreased value of *C_d_* obtains higher frequencies for each fixed combination of *K_W_*, *K_G_*.

**Figure 7:**
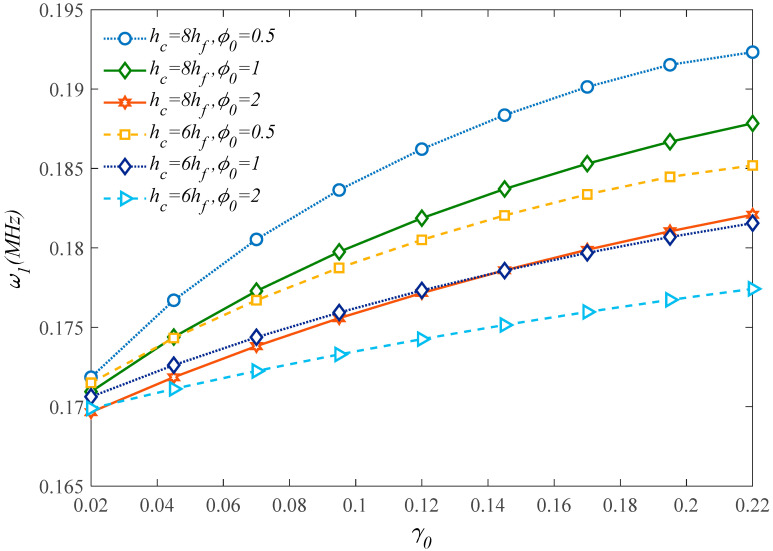
Effect of the honeycomb cells’ geometrical parameters on the first natural frequency of the structure.

The effect of the internal aspect ratio *ϕ*_0_ and dimensionless cell thickness *γ*_0_ on the first natural frequency of the sandwich microplate is plotted in Figure 7. Based on the results in this figure, larger magnitudes of *γ*_0_ lead to an increased system stability. On the other hand, a clear reduction in the structural stiffness and frequency is gained by internal aspect ratio enhancement and honeycomb core thickness reduction in the case of fixed internal cells angle equal to 30°. This means that, for a constant value of total thickness, a lower face sheet thickness to core thickness ratio results in a higher stiffness and weaker flexibility.

**Figure 8:**
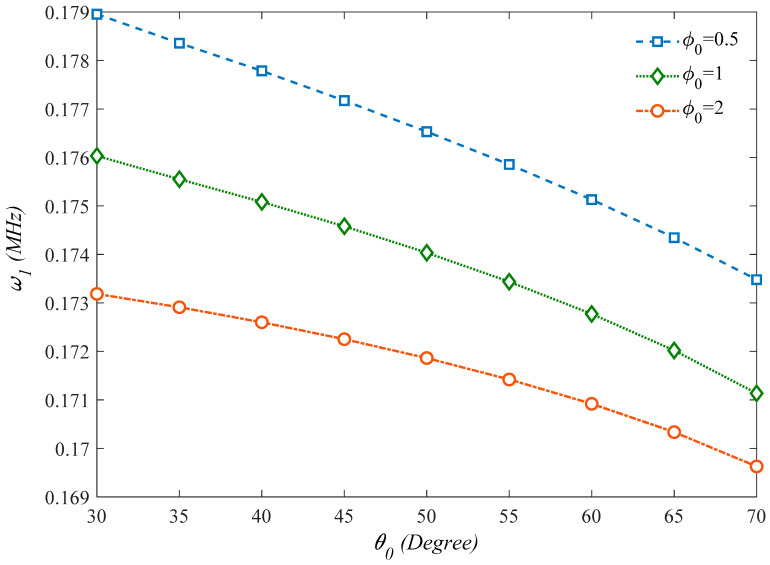
Effect of the internal cells angle of the honeycomb core on the first natural frequency of the structure (*γ*_0_ = 0.1).

**Figure 9:**
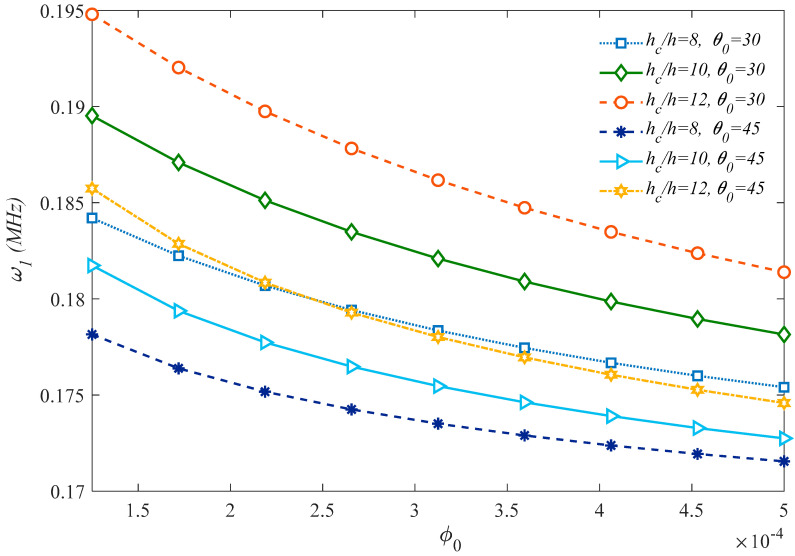
Effect of the thickness ratio on the first natural frequency of the structure.

Moreover, based on the curvatures plotted in Figure 8 and Figure 9 which represent the first natural frequency versus the honeycomb core internal cell angle *ϕ*_0_ for different internal aspect ratios *φ*_0_, it seems that an enhancement of both parameters gets a natural frequency reduction. In addition, Figure 9 illustrates that the thicker honeycomb core provides higher structural stiffness and natural frequency. As a final parametric investigation, we check for the variation of the first natural frequency with the *l_m_*/*h*, based on the MCST or CET, under the assumption of three different core thicknesses. 

**Figure 10:**
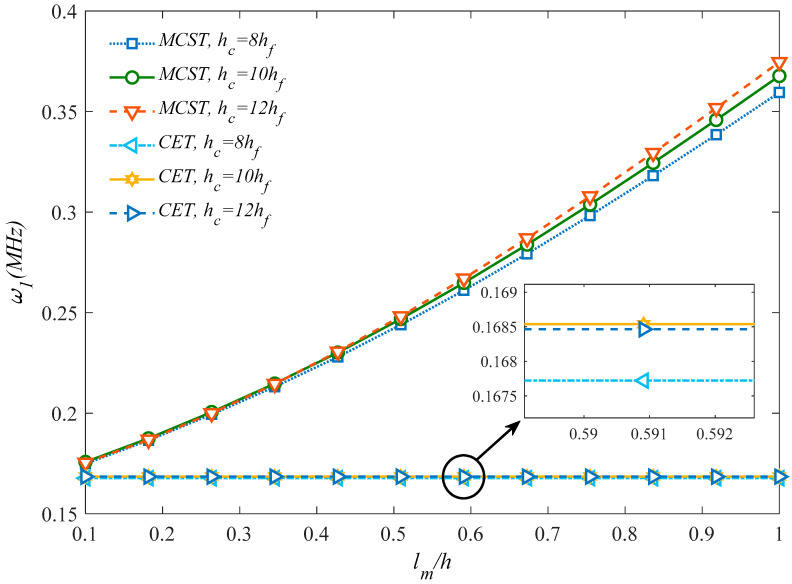
Comparison between results from a MCST and CET for different dimensionless length-scale parameters.

Based on the plots in Figure 10, it should be noted that the natural frequency increases significantly for higher values of *l_m_*/*h* ratio, when the problem is tackled by a MCST, whereas it remains almost unaffected by *l_m_*/*h* according to a CET. This confirms, once again, the great importance of adopting a size-dependent approach instead of classical formulations.

## 6. Conclusions 

In this work, a QHSDT is employed to investigate the vibrational behavior of sandwich honeycomb microplates with two GPLs’ composite face sheets, resting on elastic foundations. The equations of motion are obtained by applying the Hamilton’s principle, where the Navier-type solutions are determined in analytical form. Based on a large systematic investigation, it is noted that an Epoxy/Nomex honeycomb core makes the sandwich structure less flexible than Epoxy/Glass phenolic and uniform glass phenolic core materials, whereby a uniform Nomex honeycomb core provides the highest structural stiffness. Moreover, a larger dimensionless cell thickness (*γ_0_*) yields an increased stability in the system, whereas internal aspect ratio elevation provides structural stability reduction along with the system’s stiffness and natural frequency. The results based on a MCST are compared to predictions from CET to provide a clear understanding about vibrational responses’ sensitivity to size-dependent parameters. In agreement with findings from the literature, a CET always produces more conservative results compared to an MCST, which justifies the necessity of adopting non classical approaches instead of the classical ones. The proposed model together with our numerical results could be very useful for the design and manufacturing of many aerospace, automotive or shipbuilding engineering applications, where honeycomb structures are recommended for their great capability to tolerate high pressures and stresses despite their light structure.

## Figures and Tables

**Table 1:** Graphene nanoplatelets (GPLs) gradient index for different values of their total content [36].

Total GPLs Content (Percentage)	*λ_U_*	*λ_L_*	*λ_P_*
0	0	0	0
1/3	1/3	2/3	1
1	1	2	3

**Table 2:** Comparative evaluation between our results and those ones of Ref. [45] for a square microplate and different mode numbers.

*a*/*h*			*l_m_*/*h*					
			0.0	0.2	0.4	0.6	0.8	1.0
**5**	**Epoxy**	**Present**	0.2145	0.2322	0.2786	0.3319	0.4143	0.4815
		Ref. [45]	0.2148	0.2301	0.2708	0.3271	0.3920	0.4615
	**Uniform**	**Present**	0.4460	0.4820	0.5794	0.7114	0.8622	1.0220
		Ref. [45]	0.4468	0.4789	0.5639	0.6813	0.8164	0.9613
**10**	**Epoxy**	**Present**	0.0586	0.0632	0.0752	0.0918	0.1109	0.1315
		Ref. [45]	0.0586	0.0629	0.0745	0.0905	0.1091	0.1290
	**Uniform**	**Present**	0.1219	0.1314	0.1564	0.1910	0.2308	0.2736
		Ref. [45]	0.1219	0.1310	0.1551	0.1885	0.2271	0.2686

**Table 3:** Effect of the internal cells angle of the honeycomb core on the structural response, as predicted by modified couple stress theory (MCST) and classical elasticity theory (CET).

		*ω* (*MHz*)		
	(*m*, *n*)	θ=30°	θ=45°	θ=60°
**MCST**	**(1, 1)**	0.1789	0.1762	0.1728
	**(2, 1)**	0.3821	0.3691	0.3542
	**(2, 2**)	0.5113	0.4940	0.4758
**CET**	**(1, 1)**	0.1678	0.1633	0.1577
	**(2, 1)**	0.3555	0.3376	0.3166
	**(2, 2)**	0.4545	0.4310	0.4064

**Table 4:** Effect of the GPLs dispersion patterns on the natural frequencies of the micro structure, for different mode numbers.

	*ω* (*MHz*)			
(*m*, *n*)	Uniform (*λ_U_* = 1)	Parabolic (*λ_P_* = 1)	Linear (*λ_L_* = 2)	Epoxy
(1, 1)	0.1745	0.1637	0.2115	0.1044
(2, 1)	0.3584	0.3368	0.4246	0.2192
(2, 2)	0.4832	0.4521	0.5670	0.2950
(3, 1)	0.5849	0.5505	0.6799	0.3656
(3, 2)	0.6794	0.6378	0.7860	0.4287
(3, 3)	0.8131	0.7606	0.9387	0.5016

## Appendix

The coefficients in Equations (37)–(41) are defined as in the following

$$C_{110},C_{111},C_{112}=\int_{\frac{h_{c}}{2}}^{\frac{h_{c}}{2}+h_{t}}\text{​}C_{11f}\left(z\right)\left(1,\text{​}z,z^{2}\right)dz+\int_{-\frac{h_{c}}{2}}^{\frac{h_{c}}{2}}\text{​}C_{11c}\left(z\right)\left(1,\text{​}z,z^{2}\right)dz+\int_{-\frac{h_{c}}{2}-h_{b}}^{-\frac{h_{c}}{2}}\text{​}C_{11f}\left(z\right)\left(1,\text{​}z,z^{2}\right)dz,$$

$$C_{120},C_{121},C_{122}=\int_{\frac{h_{c}}{2}}^{\frac{h_{c}}{2}+h_{t}}\text{​}C_{12f}\left(z\right)\left(1,\text{​}z,z^{2}\right)dz+\int_{-\frac{h_{c}}{2}}^{\frac{h_{c}}{2}}\text{​}C_{12c}\left(z\right)\left(1,\text{​}z,z^{2}\right)dz+\int_{-\frac{h_{c}}{2}-h_{b}}^{-\frac{h_{c}}{2}}\text{​}C_{12f}\left(z\right)\left(1,\text{​}z,z^{2}\right)dz,$$

$$C_{220},C_{221},C_{222}=\int_{\frac{h_{c}}{2}}^{\frac{h_{c}}{2}+h_{t}}\text{​}C_{22f}\left(z\right)\left(1,\text{​}z,z^{2}\right)dz+\int_{-\frac{h_{c}}{2}}^{\frac{h_{c}}{2}}\text{​}C_{22c}\left(z\right)\left(1,\text{​}z,z^{2}\right)dz+\int_{-\frac{h_{c}}{2}-h_{b}}^{-\frac{h_{c}}{2}}\text{​}C_{22f}\left(z\right)\left(1,\text{​}z,z^{2}\right)dz,$$

$$C_{440},C_{441},C_{442}=\int_{\frac{h_{c}}{2}}^{\frac{h_{c}}{2}+h_{t}}\text{​}C_{44f}\left(z\right)\left(1,\text{​}z,z^{2}\right)dz+\int_{-\frac{h_{c}}{2}}^{\frac{h_{c}}{2}}\text{​}C_{44c}\left(z\right)\left(1,\text{​}z,z^{2}\right)dz+\int_{-\frac{h_{c}}{2}-h_{b}}^{-\frac{h_{c}}{2}}\text{​}C_{44f}\left(z\right)\left(1,\text{​}z,z^{2}\right)dz,$$

$$F_{110},F_{111},F_{112}=\int_{\frac{h_{c}}{2}}^{\frac{h_{c}}{2}+h_{t}}\text{​}C_{11f}\left(z\right)f\left(z\right)\left(1,\text{​}z,f\left(z\right)\right)dz+\int_{-\frac{h_{c}}{2}}^{\frac{h_{c}}{2}}\text{​}C_{11c}\left(z\right)f\left(z\right)\left(1,\text{​}z,f\left(z\right)\right)dz+\int_{-\frac{h_{c}}{2}-h_{b}}^{-\frac{h_{c}}{2}}\text{​}C_{11f}\left(z\right)f\left(z\right)\left(1,\text{​}z,f\left(z\right)\right)dz,$$

$$F_{120},F_{121},F_{122}=\int_{\frac{h_{c}}{2}}^{\frac{h_{c}}{2}+h_{t}}\text{​}C_{12f}\left(z\right)f\left(z\right)\left(1,\text{​}z,f\left(z\right)\right)dz+\int_{-\frac{h_{c}}{2}}^{\frac{h_{c}}{2}}\text{​}C_{12c}\left(z\right)f\left(z\right)\left(1,\text{​}z,f\left(z\right)\right)dz+\int_{-\frac{h_{c}}{2}-h_{b}}^{-\frac{h_{c}}{2}}\text{​}C_{12f}\left(z\right)f\left(z\right)\left(1,\text{​}z,f\left(z\right)\right)dz,$$

$$F_{220},F_{221},F_{222}=\int_{\frac{h_{c}}{2}}^{\frac{h_{c}}{2}+h_{t}}\text{​}C_{22f}\left(z\right)f\left(z\right)\left(1,\text{​}z,f\left(z\right)\right)dz+\int_{-\frac{h_{c}}{2}}^{\frac{h_{c}}{2}}\text{​}C_{22c}\left(z\right)f\left(z\right)\left(1,\text{​}z,f\left(z\right)\right)dz+\int_{-\frac{h_{c}}{2}-h_{b}}^{-\frac{h_{c}}{2}}\text{​}C_{22f}\left(z\right)f\left(z\right)\left(1,\text{​}z,f\left(z\right)\right)dz,$$

$$F_{440},F_{441},F_{442}=\int_{\frac{h_{c}}{2}}^{\frac{h_{c}}{2}+h_{t}}\text{​}C_{44f}\left(z\right)f\left(z\right)\left(1,\text{​}z,z^{2}\right)dz+\int_{-\frac{h_{c}}{2}}^{\frac{h_{c}}{2}}\text{​}C_{44c}\left(z\right)f\left(z\right)\left(1,\text{​}z,z^{2}\right)dz+\int_{-\frac{h_{c}}{2}-h_{b}}^{-\frac{h_{c}}{2}}\text{​}C_{44f}\left(z\right)f\left(z\right)\left(1,\text{​}z,z^{2}\right)dz,$$

$$E_{130},E_{131},E_{132}=\int_{\frac{h_{c}}{2}}^{\frac{h_{c}}{2}+h_{t}}\text{​}C_{13f}\left(z\right)g^{\prime }\left(z\right)\left(1,\text{​}z,f\left(z\right)\right)dz+\int_{-\frac{h_{c}}{2}}^{\frac{h_{c}}{2}}\text{​}C_{13c}\left(z\right)g^{\prime }\left(z\right)\left(1,\text{​}z,f\left(z\right)\right)dz+\int_{-\frac{h_{c}}{2}-h_{b}}^{-\frac{h_{c}}{2}}\text{​}C_{13f}\left(z\right)g^{\prime }\left(z\right)\left(1,\text{​}z,f\left(z\right)\right)dz,$$

$$E_{320},E_{321},E_{322}=\int_{\frac{h_{c}}{2}}^{\frac{h_{c}}{2}+h_{t}}\text{​}C_{32f}\left(z\right)g^{\prime }\left(z\right)\left(1,\text{​}z,f\left(z\right)\right)dz+\int_{-\frac{h_{c}}{2}}^{\frac{h_{c}}{2}}\text{​}C_{32c}\left(z\right)g^{\prime }\left(z\right)\left(1,\text{​}z,f\left(z\right)\right)dz+\int_{-\frac{h_{c}}{2}-h_{b}}^{-\frac{h_{c}}{2}}\text{​}C_{32f}\left(z\right)g^{\prime }\left(z\right)\left(1,\text{​}z,f\left(z\right)\right)dz,$$

$$E_{330}=\int_{\frac{h_{c}}{2}}^{\frac{h_{c}}{2}+h_{t}}C_{33f}\left(z\right)g^{\prime }{\left(z\right)}^{2}dz+\int_{-\frac{h_{c}}{2}}^{\frac{h_{c}}{2}}C_{33c}\left(z\right)g^{\prime }{\left(z\right)}^{2}dz+\int_{-\frac{h_{c}}{2}-h_{b}}^{-\frac{h_{c}}{2}}C_{33f}\left(z\right)g^{\prime }{\left(z\right)}^{2}dz,$$

$$G_{550},G_{551}=\int_{\frac{h_{c}}{2}}^{\frac{h_{c}}{2}+h_{t}}\text{​}C_{55f}\left(z\right)g\left(z\right)\left(1,\text{​}f^{\prime }\left(z\right)\right)dz+\int_{-\frac{h_{c}}{2}}^{\frac{h_{c}}{2}}\text{​}C_{55c}\left(z\right)g\left(z\right)\left(1,\text{​}f^{\prime }\left(z\right)\right)dz+\int_{-\frac{h_{c}}{2}-h_{b}}^{-\frac{h_{c}}{2}}\text{​}C_{55f}\left(z\right)g\left(z\right)\left(1,\text{​}f^{\prime }\left(z\right)\right)dz,$$

$$G_{660},G_{661}=\int_{\frac{h_{c}}{2}}^{\frac{h_{c}}{2}+h_{t}}\text{​}C_{66f}\left(z\right)g\left(z\right)\left(1,\text{​}f^{\prime }\left(z\right)\right)dz+\int_{-\frac{h_{c}}{2}}^{\frac{h_{c}}{2}}\text{​}C_{66c}\left(z\right)g\left(z\right)\left(1,\text{​}f^{\prime }\left(z\right)\right)dz+\int_{-\frac{h_{c}}{2}-h_{b}}^{-\frac{h_{c}}{2}}\text{​}C_{66f}\left(z\right)g\left(z\right)\left(1,\text{​}f^{\prime }\left(z\right)\right)dz,$$

$$K,K_{0},K_{1},K_{2},K_{3},K_{4},K_{5}=\int_{\frac{h_{c}}{2}}^{\frac{h_{c}}{2}+h_{t}}\text{​}l_{m}{}^{2}\mu_{f}\left(z\right)(1,\text{​}g\left(z\right),f^{\prime }\left(z\right),g\left(z\right)f^{\prime }\left(z\right),f^{\prime }{\left(z\right)}^{2},g^{\prime }\left(z\right)f^{\prime \prime }\left(z\right),f^{\prime \prime }\left(z{)}^{2}\right)dz+\;\;\;\;\;\;\;\;\;+\int_{-\frac{h_{c}}{2}}^{\frac{h_{c}}{2}}\text{​}l_{m}{}^{2}\mu_{c}\left(z\right)(1,\text{​}g\left(z\right),f^{\prime }\left(z\right),g\left(z\right)f^{\prime }\left(z\right),f^{\prime }{\left(z\right)}^{2},g^{\prime }\left(z\right)f^{\prime \prime }\left(z\right),f^{\prime \prime }\left(z{)}^{2}\right)dz+\;\;\;\;\;\;\;\;\;\;+\int_{-\frac{h_{c}}{2}-h_{b}}^{-\frac{h_{c}}{2}}\text{​}l_{m}{}^{2}\mu_{f}\left(z\right)(1,\text{​}g\left(z\right),f^{\prime }\left(z\right),g\left(z\right)f^{\prime }\left(z\right),f^{\prime }{\left(z\right)}^{2},g^{\prime }\left(z\right)f^{\prime \prime }\left(z\right),f^{\prime \prime }\left(z{)}^{2}\right)dz,$$

$$I_{0},I_{1},I_{2},I_{3},I_{4},I_{5},I_{6},I_{7}=\overset{\frac{h_{c}}{2}+h_{t}}{\underset{\frac{h_{c}}{2}}{\int }}\text{​}\rho_{f}\left(z\right)(1,\text{​}z,z^{2},f\left(z\right),g\left(z\right),zf\left(z\right),f{\left(z\right)}^{2},g\left(z{)}^{2}\right)dz+\;\;\;\;\;\;\;+\overset{\frac{h_{c}}{2}}{\underset{-\frac{h_{c}}{2}}{\int }}\text{​}\rho_{c}(1,\text{​}z,z^{2},f\left(z\right),g\left(z\right),zf\left(z\right),f{\left(z\right)}^{2},g\left(z{)}^{2}\right)dz+\;\;\;\;\;\;\;\;+\overset{-\frac{h_{c}}{2}}{\underset{-\frac{h_{c}}{2}-h_{b}}{\int }}\text{​}\rho_{f}\left(z\right)(1,\text{​}z,z^{2},f\left(z\right),g\left(z\right),zf\left(z\right),f{\left(z\right)}^{2},g\left(z{)}^{2}\right)dz$$
